# Relaxin 2/RXFP1 Signaling Induces Cell Invasion via the β-Catenin Pathway in Endometrial Cancer

**DOI:** 10.3390/ijms19082438

**Published:** 2018-08-18

**Authors:** Misaki Fue, Yasuhiro Miki, Kiyoshi Takagi, Chiaki Hashimoto, Nobuo Yaegashi, Takashi Suzuki, Kiyoshi Ito

**Affiliations:** 1 Department of Disaster Obstetrics and Gynecology, International Research Institute of Disaster Science (IRIDeS), Tohoku University, Sendai 980-8575, Japan; fue@med.tohoku.ac.jp (M.F.); kito@med.tohoku.ac.jp (K.I.); 2 Department of Pathology and Histotechnology, Tohoku University Graduate School of Medicine, Sendai 980-8575, Japan; k-takagi@med.tohoku.ac.jp (K.T.); t-suzuki@patholo2.med.tohoku.ac.jp (T.S.); 3 Department of Obstetrics and Gynecology, Tohoku University Graduate School of Medicine, Sendai 980-8575, Japan; chiaki@med.tohoku.ac.jp (C.H.); nobuo.yaegashi@gmail.com (N.Y.)

**Keywords:** relaxin 2, RXFP1, phospho-β-catenin, endometrial cancer

## Abstract

Relaxin is known to play an important role in animal pregnancies, including those of humans. It is suggested that relaxin induces aggressive cell growth and invasiveness in several types of cancer, including endometrial cancer. However, the mechanisms of relaxin remain largely unclear. In this study, we examined the effects of relaxin 2 (RLN2), the major circulating relaxin in humans, on human endometrial carcinoma cell lines. RLN2 treatment induced invasion in HEC-1B and Ishikawa cells. RLN2-induced cell invasion was significantly decreased by transfection of relaxin receptor 1 (RXFP1) siRNAs. The β-catenin inhibitor, XAV939, also significantly inhibited the RLN2-induced cell invasions. Both a decrease of cadherin expression and an increase of β-catenin phosphorylation were observed in response to the RLN2 treatment in HEC-1B and Ishikawa cells. We then examined RLN2 and RXFP1 expression in 80 human endometrioid endometrial carcinoma tissues. RLN2 immunoreactivity was detected in the human endometrial carcinoma cells and had a correlative tendency with histological grade and RXFP1. These results suggest that adherens junctions in cancer cells are weakened by the breakdown of the cadherin/catenin complex, which is induced by β-catenin phosphorylation via RLN2/RXFP1 signaling.

## 1. Introduction

Endometrial carcinoma is the most common gynecological malignancy of the female genital tract and, recently, its incidence has been increasing [1,2], especially that of endometrioid endometrial adenocarcinoma, which is the most common histological type. In most cases, patients are diagnosed with stages I and II carcinoma, however those that are diagnosed with advanced stages have a poor prognosis. The overall 5-year survival rate is 80.1% for stage III and 13.3% for stage IV, respectively [3]. Moreover, 10–17% of patients experience recurrence even when they are diagnosed at an early stage, suggesting that current therapies are not particularly effective [4,5,6]. Thus, it is very important to understand the mechanism of disease progression to correctly predict the recurrence of the condition in patients with endometrial carcinomas.

The insulin-related peptide hormone relaxin (RLN) is known as a central pregnancy hormone [7]. It is also known to affect the brain, kidneys, connective tissue, reproductive system, and cardiovascular system through G protein-coupled receptors, such as relaxin family peptide 1 (RXFP1) and 2 (RXFP2), which activate cyclic adenosine monophosphate in response to RLN binding [8]. Relatively high levels of RXFP1 are reported in uterine tissue [9,10,11]. It is well-known that three peptide forms of RLN: RLN1, RLN2, and RLN3, exist in humans, although the roles of two of the peptides, RLN1 and RLN3, remain unclear [12]. RLN2, which is produced in male prostate tissue [13], and female corpus luteum tissue [14,15] is known as a major human circulating RLN [12,16]. The effects of RLN have been established in female reproduction and pregnancy. It is considered that the physiological roles of RLN are the maintenance of the pregnancy and supportive effects during delivery. Particularly in late pregnancy, RLN increases pelvic girdle relaxation, which is important to secure the passage of the fetus [17]. RLX is also known to have inhibitory effects on the proliferation of cardiac fibroblasts and the synthesis of collagen [18]. Therefore, it is suggested that RLN plays an important role in heart failure.

RLN is present in breast cancer tissues and is associated with carcinoma cell growth, motility, adhesion, and in vitro invasiveness [19,20,21,22]. RLN treatment increases both in vitro migration and invasiveness of breast and thyroid cancer cells through the up-regulation of matrix metalloproteinase (MMP) activity and vascular endothelial growth factor expression [11,21,22]. In endometrial cancer, RLN increases MMP-2 or MMP-9 expression by binding to RXFP1 [11]. Furthermore, Thompson et al. [23] reported that RLN up-regulated the Protocadherin Y (PCDHY)/Wnt signal in prostate cancer cells, affecting the stability of β-catenin. It is well established that E-cadherin is degraded by tyrosine phosphorylation of β-catenin in invasive cancers with a high metastatic ability. The loss of E-cadherin has been well-known to promote migration and invasiveness in several cancer cell types, including endometrial cancer [24,25,26]. These findings suggest that the Wnt/β-catenin signaling pathway is the key pathway that is induced by RLN-RXFP to enable endometrial cancer invasion.

However, the biological and clinical significance of RLN in endometrial cancer remains unclear. Therefore, in this study, we first examined the effects of RLN2 on Ishikawa and HEC-1B human endometrial cancer cell line invasiveness. We then investigated the role of RLN2-induced β-catenin phosphorylation on cell invasiveness and E-cadherin degradation in both Ishikawa and HEC-1B cells. Furthermore, the significance of RLN2 and RXFP1 was examined by immunohistochemistry in 80 cases of endometrioid endometrial carcinoma (EEC) tissues. 

## 2. Results

### 2.1. Immunohistochemistry

**Figure 1:**
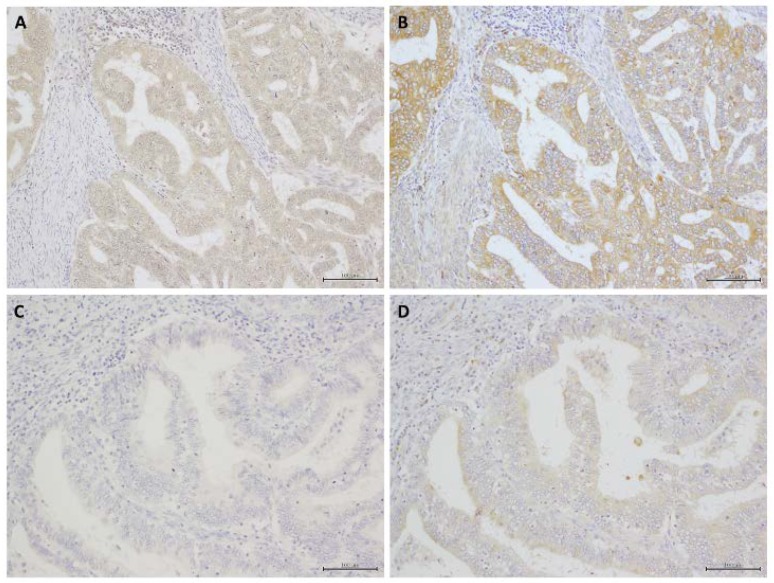
Immunohistochemistry for RXFP1 and RLN2 in human endometrial carcinomas. (**A**) RXFP1-high case, (**B**) RLN2-high case, (**C**) RXFP1-low case, and (**D**) RLN2-low case. Scale bar, 100 μm.

RXFP1 immunoreactivity was detected in the cytoplasmic membrane of carcinoma cells, and RLN2 immunoreactivity was detected in the cytoplasm of carcinoma cells (Figure 1). The associations between RLN2 immunoreactivity and clinicopathological parameters are summarized in Table 1. Of the 80 cases of EEC tissues that were examined, 54 (68%) were positive for RLN2 immunoreactivity, and 43 (54%) were positive for RXFP1 immunoreactivity. RLN2 expression had a slightly positive association with histological grade (*p* = 0.0856) and RXFP1 expression (*p* = 0.0570) in EEC (Table 1).

### 2.2. Effect of RLN2 on HEC-1B and Ishikawa Cell Invasiveness

**Figure 2:**
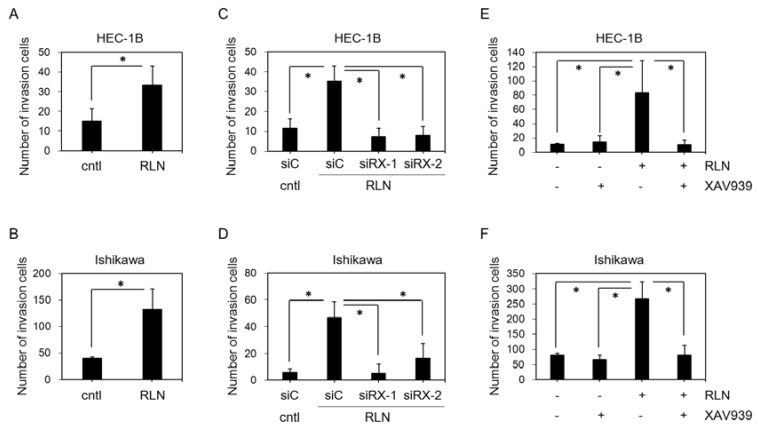
(**A**,**B**) The effect of relaxin 2 on the invasion of HEC-1B (**A**) and Ishikawa (**B**) cells. HEC-1B and Ishikawa cells were treated with RLN for 24 h. (**C**,**D**) The effect of down-regulated RXFP1 on invasion, which was induced by relaxin 2 treatment, in HEC-1B (**C**) and Ishikawa (**D**). (**E**,**F**) The effect of XAV939 on RLN induced-invasion of HEC-1B (**E**) and Ishikawa (**F**) cells. HEC-1B and Ishikawa cells treated with RLN alone or combined with XAV939 for 24 h. The data are expressed as means ± SD of three independent experiments with technical triplicates for each. *, *p* < 0.05 (Student’s *t*-test). cntl, vehicle control; RLN, relaxin 2; siC, control siRNA; siRX, RXFP1 siRNA.

The invasive potential of HEC-1B (Figure 2A) and Ishikawa (Figure 2B) cells following RLN2 stimulation was explored. RLN2 significantly enhanced the invasion of both HEC-1B (*p* = 0.03) and Ishikawa (*p* = 0.007) cells through the Matrigel-coated membrane.

### 2.3. Effect of siRXFP1 on HEC-1B and Ishikawa Cell Invasiveness

RXFP1 immunoreactivities that were detected by immunofluorescence staining were markedly diminished by the two different siRNA constructs targeting RXFP1 (siRXFP1-1 and -2) in both the HEC-1B (Supplementary Figure S1) and Ishikawa cells (Supplementary Figure S2), respectively. 

In HEC-1B which was transfected with control siRNA (siC), the RLN2 treatment significantly increased cell invasiveness (*p* = 0.005). The RLN2-mediated induction of HEC-1B invasion was significantly inhibited by the transfection of both siRXFP1-1 (*p* = 0.002) and -2 (*p* = 0.003) (Figure 2C). In the Ishikawa cells, cell invasiveness was significantly increased by RLN2 treatment (*p* = 0.002) and was significantly suppressed by siRXFP1-1 (*p* = 0.003) and -2 (0.016) (Figure 2D).

### 2.4. Effect of XAV939 on HEC-1B and Ishikawa Cell Invasiveness

In both the HEC-1B (Figure 2E) and Ishikawa (Figure 2F) cells, the RLN2 treatment significantly increased the number of invasive cells (HEC-1B, *p* = 0.03; Ishikawa, *p* = 0.002). This RLN2-mediated cell invasion was significantly inhibited by β-catenin inhibitor (XAV9399) [27] treatment (HEC-1B, *p* = 0.03; Ishikawa, *p* = 0.004). XAV939 alone did not significantly change HEC-1B (*p* = 0.27) and Ishikawa (*p* = 0.09) invasiveness.

### 2.5. Effect of RLN2 on β-Catenin Phosphorylation in Cancer Cell Lines

**Figure 3:**
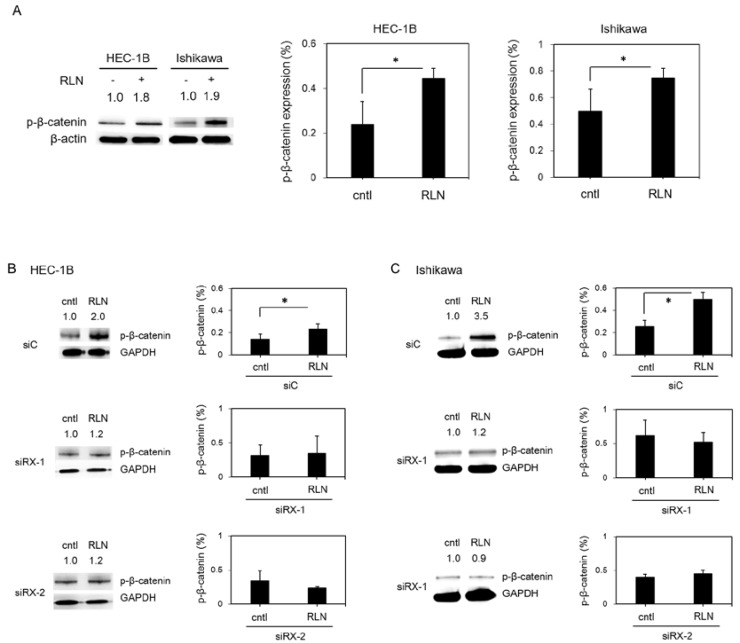
(**A**) The effect of RLN on β-catenin phosphorylation in HEC-1B and Ishikawa cells. Cells were stimulated with RLN for 24 h. (**B**,**C**) The effect of down-regulated RXFP1 on β-catenin phosphorylation, which was induced by relaxin 2 treatment, in HEC-1B (**B**) and Ishikawa (**C**). Relative intensity values, which have been normalized to the values for the control (− = 1.0), are shown. The data are expressed as means ± SD of three independent experiments with technical triplicates for each. *, *p* < 0.05 (Student’s *t*-test). cntl, vehicle control; RLN, relaxin 2; siC, control siRNA; siRX, RXFP1 siRNA. p-β-catenin, phospho β-catenin.

After incubation with RLN2 for 24 h, total protein lysates were prepared and analyzed by western blot. The expression levels of the total p-β-catenin increased in both the HEC-1B (*p* = 0.02) and Ishikawa (*p* = 0.04) cells (Figure 3A). 

The suppression of RXFP1 expression by siRNAs was associated with a significant decrease in the total p-β-catenin expression, which was induced by RLN2 treatment, in the HEC-1B (siC, *p* = 0.005; siRXFP1-1, *p* = 0.43; and siRXFP1-2, *p* = 0.14) (Figure 3B) and Ishikawa cells (siC, *p* = 0.004; siRXFP1-1, *p* = 0.28; and siRXFP1-2, *p* = 0.14) (Figure 3C).

### 2.6. Effect of RLN2 on Cadherin Expression in HEC-1B and Ishikawa Cells 

**Figure 4:**
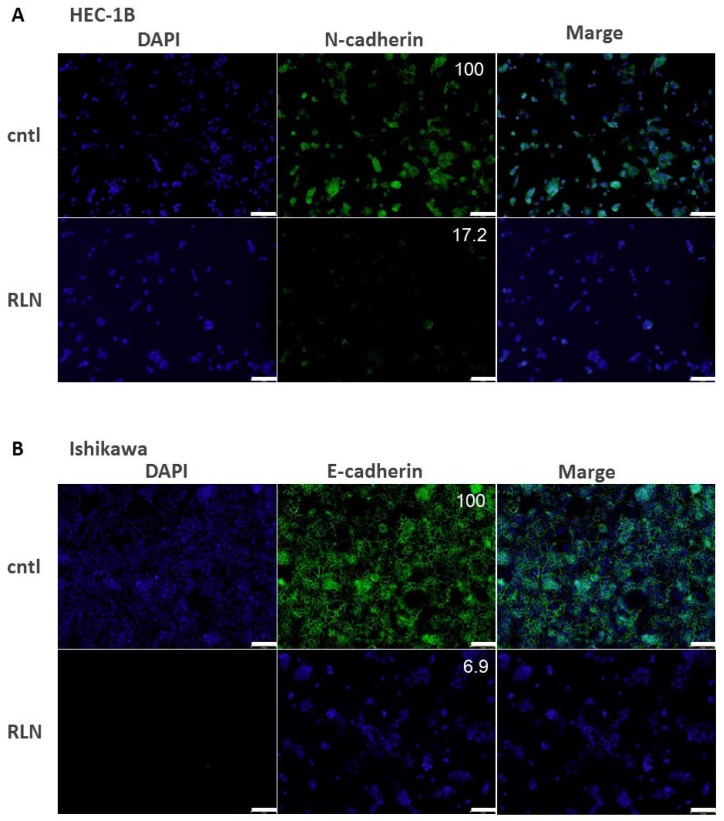
(**A**,**B**) Cadherin expression following 24 h of RLN2 stimulation in HEC-1B (**A**) and Ishikawa (**B**) cells. Cadherins were labeled with Alexa Fluor 488 (green). The nuclear was labeled with DAPI (blue). The relative intensity values, which were normalized to the values for the control (cntl = 100), are shown. cntl, vehicle control; RLN, relaxin 2. Scale bar, 10 μm.

After incubation with RLN2 for 24 h, the expression levels of N-cadherin or E-cadherin in HEC-1B and Ishikawa cells were examined by immunofluorescence cytochemistry. N-cadherin was detected in HEC-1B cells, and E-cadherin was detected in Ishikawa cells. The N- and E-cadherin expression levels decreased in response to RLN2 stimulation (Figure 4).

## 3. Discussion

This is the first study addressing RLN2-induced cellular invasion through RXFP1 signal transduction, β-catenin phosphorylation, and loss of E-cadherin in in vitro endometrial cancer cells. We demonstrated the significance of RXFP1 through immunohistochemistry in EEC tissues by first showing that RXFP1 immunoreactivity was detected in 54% of the EEC tissues that were examined, and that it positively correlated with RLN2 expression. Furthermore, RLN2 expression positively correlated with the histological grade.

Kamat et al. [11] reported the effect of RXFP siRNA on the expression of MMP in HEC-B and KLE endometrial cancer cell lines. In their reports, porcine RLN induced the expression of MMP-2 and MMP-9 in HEC-1B and KLE cells, respectively [11,28]. In our present study, RLN2 treatment induced HEC-1B and Ishikawa cell invasiveness, and RXFP1 siRNA transfection significantly decreased it. The function of porcine RLN is similar to that of recombinant human RLN and it had previously been used in clinical trials for pregnant women [29,30,31]. However, it is also true that there is a structural difference between porcine and human RLN [32]. Further examination regarding the expression of invasiveness-related factors will be required to clarify the mechanisms of invasion in RLN2-treated human endometrial cancer cells. 

In our present study, RLN2 treatment significantly increased p-β-catenin expression in both HEC-1B and Ishikawa cells. These increases in p-β-catenin were significantly inhibited by siRXFP1-1 and -2 transfection. In addition, after co-treatment with XAV939 and RLN2, RLN2-induced invasion significantly decreased in both HEC-1B and Ishikawa cells. This suggests that RLN2 is a potent signal for β-catenin phosphorylation via RXFP1 binding in endometrial cancer cells. Otherwise, the invasive ability was very low for the control HEC-1B cells of intact status. Therefore, XAV939 alone did not exert an inhibitory effect on the invasiveness of the HEC-1B cells. In the Ishikawa cells, the invasive ability was relatively high compared to that of the HEC-1B cells. Therefore, invasion tended to be slightly suppressed by treatment with XAV939 alone. RLN has previously been shown to regulate the PCDHY/Wnt pathway in prostate cancer cells [11]. PCDHY is known to interact with β-catenin and to induce the Wnt signal in prostate cancer cells [33]. It is also known that β-catenin accumulates in the cytoplasm through Wnt binding to its ligands [34]. Elevated cytosolic β-catenin then translocates to the nucleus and exerts genomic effects, including the transcription of invasion-related factors [35]. In several types of cancer, including endometrial cancer, β-catenin is related to cancer metastasis [36]. It has been reported that catenins, which bind to the region of the E-cadherin cytoplasmic domain, are required for cell-cell adhesion [37,38,39], however the cell-cell adhesion function is maintained when the phosphorylation of β-catenin is suppressed [40]. In this study, we demonstrated that RLN2 strongly reduced N- and E-cadherin expression in HEC-1B and Ishikawa cells, respectively. It has previously been reported in HEC-1B cells that the E-cadherin gene is suppressed in the promoter region by 5′ CpG island methylation [41]. An important hallmark to use in the differential diagnosis of RLN2-induced invasive endometrial cancer is β-catenin phosphorylation via RXFP1 and a weakening of the adherens junctions through breakage of the cadherin/catenin complex. 

It was previously reported that RLN2 immunoreactivity was positively correlated with histological grade and myometrial invasion in tissue microarray samples from 57 endometrial cancer cases [11]. In our study, RLN2 immunoreactivity was marginally associated with histological grade in endometrial carcinoma cases. These findings suggest that RLN2 is involved in endometrial cancer malignancy and tumor progression. It was also reported that RXFP1 mRNA is expressed in endometrial cancer tissues [11], however its influence on cancer cell behavior remained unclear. Both our in vitro analysis and previous report [11] demonstrated that RXFP1 had a crucial role in RLN2-induced cancer invasion. In this study, we first demonstrated the expression of RXFP1 in endometrial cancer tissues. However, there was no significant association between RXFP1 immunoreactivity and clinicopathological parameters in the endometrial cancer cases that were examined. It is true that RLN2 is known to bind to RXFP2 with high affinity, as well as to RXFP1 [42]. Future studies will require clarifying the role of RLN2 and its receptor signaling in endometrial cancer cells. RLN2 is known to cross-react with RXFP2 [43], however there is no information about the biological function of RLN2 via RXFP2 in human cells [44]. Therefore, further examination of the expression profile of the relaxin family and their corresponding receptors is required to clarify the role of relaxin signaling in endometrial cancer. In addition, the serum RLN2 or relaxin level has been well examined and is reported to have some benefit as a predictive marker for malignant features, such as metastasis, and for prognosis for several types of cancer, including prostate [45], ovarian [46], esophageal [47], breast [48], and osteosarcoma [49]. In this study, we did not have any serum samples for the measurement of RLN2 concentration. However, our results suggest that RLN2 could be evaluated by immunohistochemistry using FFPE samples for pathological diagnosis. Further examination regarding the correlation between the serum or intratumoral RLN2 concentration and the immunohistochemical status of RLN2 is necessary to use RLA2 evaluation for clinical predictions or as a prognostic marker for endometrial cancer. 

## 4. Materials and Methods

### 4.1. Patients

A total of 80 endometrial endometrioid adenocarcinoma tissues of postmenopausal women were retrieved from the surgical pathology files of Tohoku University Hospital, Sendai, Japan. The Ethics Committee of Tohoku University School of Medicine approved the research protocol of this examination. None of the patients received radiation therapy, hormone therapy, or chemotherapy prior to surgery. The operative treatment methods in Tohoku University Hospital were summarized in our previous study [50]. The study was approved by the Tohoku University Graduate School of Medicine Research Ethical Committee (No. 2013-1-265, 17 September 2013).

### 4.2. Immunohistochemistry

Anti-RLN2 antibody (ab183505) was purchased from Abcam (Cambridge, UK) and anti-RXFP1 antibody (GTX12714) was purchased from GeneTex (Irvine, CA, USA). A Histofine Kit (Nichirei Bioscience, Tokyo, Japan), which is based on the streptavidin-biotin amplification method, was used in the present study. In this study, we employed formalin-fixed paraffin-embedded tissues for pathological diagnoses. Tissue samples, which were sectioned at a thickness of 3 µm, were deparaffinized and immersed in methanol with 0.3% hydrogen peroxide. The sections were heated in an autoclave at 121 °C for 5 min in 0.01 M citrate buffer (pH 6.0). Then, the reacted samples were incubated with the primary antibodies that were described above at 4 °C overnight. The antigen-antibody complex was visualized with 3,3′-diaminobenzidine (1 mM diamino benzidine, 50 mM Tris–HCl buffer, pH 7.6, and 0.006% H_2_O_2_) and was counterstained with hematoxylin. As a negative control, phosphate buffered saline was used instead of primary antibodies.

The immunoreactivities of RLN2 and RXFP1 were detected in the cytoplasm of the cancer cells. The cytoplasmic immunoreactivity was evaluated based on the methods from our previous reports [50,51]. For a semiquantitative evaluation of the immunohistochemistry, the intensity and areas of immunoreactivity were taken into consideration. Specimens with more than 50% immunoreactivity were defined as positive cases, and those with less than 50% immunoreactivity were judged as negative cases.

### 4.3. Cell Lines and Culture Conditions

The human endometrial cancer HEC-1B and Ishikawa cell lines were obtained from JCRB Cell Bank (Osaka, Japan). HEC-1B was cultured in Dulbecco’s Modified Eagle Medium (Gibco BRL, Palo Alto, CA, USA) and Ishikawa was cultured in MEM (Gibco). Both of the cell lines were supplemented with 10% fetal bovine serum (Biosera, Ringmer, UK). Human RLN2 (SRP3147) was purchased from Sigma-Aldrich (St. Louis, MO, USA). β-catenin inhibitor, XAV939 (#13596) was purchased from Cayman Chemical (Ann Arbor, MI, USA). Both RLN2 and XAV939 were dissolved in dimethyl sulfoxide (Wako Purechemical Industries, Osaka, Japan). The final concentrations of DMSO that were used in this study did not exceed 0.005% in any of the examined cases. The cells were treated with RLN (100 nM) or DMSO (0.005%) as a control in each experiment. XAV939 (100 nM) was simultaneously added with RLN (100 nM).

### 4.4. Matrigel Invasion Assays

The Matrigel invasion assay was conducted based on the methods from our previous study [52]. A 24-well plate and Chemotaxicell (8 µm pore size; Kurabo, Osaka, Japan) were used. The upper surface of the Chemotaxicell membrane was coated with 80 mg/cm^2^ of Matrigel basement membrane matrix (BD Bioscience, Franklin Lakes, NJ, USA). The HEC-1B and Ishikawa cells were plated in the upper chamber, and 100 nM RLN2 solution and/or 100 nM XAV939 was added to the culture plate. The cells on the upper surface of the membrane were removed after incubation for 24 h. The invasiveness was evaluated as the total number of cells on the lower surface of the membrane.

### 4.5. Small Interfering RNA Transfection 

Small interfering RNA for RXFP1 was purchased from Takara Bio (Shiga, Japan). The target sequences of siRNA against RXFP1 were as follows: siRNA-1, 5′-GGACUGAAUAGCCUUACUATT-3′ (sense), and 3′-TT CCUGACUUAUCGGAAUGAU-5′ (anti-sense); and siRNA-2, 5′-GCAGAACAAUUACAGUUC UTT-3′ (sense), and 3′-TT CGUCUUGUUAAUGUCAAGA-5′ (anti-sense). In addition, medium GC duplex #2 (Invitrogen, Carlsbad, CA, USA) was also used as a negative control (siC). siRNA, 10 nM, was transfected into cells (1 × 10^5^ cells/mL) using ScreenFect siRNA (Wako Purechemical Industries, Osaka, Japan) for 72 h. The procedure for the ScreenFect siRNA-mediated transfection in a 48-well culture plate was as follows. The siRNA was diluted in 16 μL of buffer, was mixed with 16 μL of buffer containing 0.8 μL ScreenFect siRNA, and was then incubated for 30 min at 25 °C before being added to each well. 

### 4.6. Immunoblotting

HEC-1B and Ishikawa protein was extracted using the RIPA Lysis Buffer System (Santa Cruz Biotechnology, Dallas, TX, USA). Five micrograms of protein (whole cell extracts) was subjected to sodium dodecyl sulfate-poly acrylamide gel electrophoresis (10% acrylamide gel). Following SDS-PAGE, proteins were transferred onto Hybond-P Polyvinylidene Difluoride membrane (GE Healthcare, Little Chalfont, UK). Anti-phospho-β-catenin antibody (#4176) was purchased from Cell Signaling Technology (Beverly, MA, USA). Anti-β-actin was purchased from Sigma, and anti- glyceraldehyde-3-phosphate dehydrogenase (GAPDH)-Horseradish peroxidase was purchased from Wako. Antibody–protein complexes were detected on the blots using ECL Plus Western blotting detection reagent (GE Healthcare), and the protein bands were visualized with a LAS-4000 image analyzer (Fuji Photo Film, Tokyo, Japan). The relative intensity values of the protein bands were measured by the use of an imaging analyzer, Lumina Vision (Mitani Corp., Fukui, Japan). The expression level for phospho-β-catenin was summarized as a ratio of GAPDH. 

### 4.7. Immunofluorescence Cytochemistry

Cells that were grown on cover slides were fixed in 10% formaldehyde. These samples were further incubated overnight at 4 °C with the following primary antibodies: E-cadherin (24E10) rabbit monoclonal antibody (Cell Signaling Technology) and N-cadherin rabbit polyclonal antibody (GeneTex). The samples were subsequently treated with fluorescence-labeled secondary antibodies (Alexa Fluor 488, Thermo Fisher Scientific, Waltham, MA, USA). The reacted slides were then mounted with mounting medium with DAPI. The relative intensity values of Alexa Fluor 488 (green) were measured by using an imaging analyzer (Lumina Vision). 

### 4.8. Statistical Analysis

The association between RLN2 and RXFP1 immunoreactivity and clinicopathological parameters was evaluated by the χ^2^-test. A Student’s *t*-test was performed for the in vitro experiments. P values that were less than 0.05 were considered statistically significant.

## Figures and Tables

**Table 1:** The association between RLN2 status and clinicopathological parameters in 80 endometrial carcinomas.

Parameter	Total (*n* = 80)	RLN2		
		+ (*n* = 54)	− (*n* = 26)	*p*-Value
Age	57 (29–85)	57 (30–85)	53.5 (29–76)	0.5153
Grade 1 (G1)	40	23	17	
2 (G2)	25	19	6	
3 (G3)	15	13	2	0.0856
Stage				
1 & 2	60	42	18	
3 & 4	20	12	8	0.3081
RXFP1				
Positive	43	33	10	
Negative	37	21	16	0.0570
Estrogen receptor				
Positive	51	32	19	
Negative	29	22	7	0.2225
Progesterone receptor				
Positive	24	13	11	
Negative	56	41	15	0.1001
Myometrial invasion *				
Positive	29	19	10	
Negative	48	33	15	0.7691

Age was presented as median (min–max). Statistical analyses were performed by χ^2^-test, and *p* < 0.05 was considered significant. *, There was no data for 2 of the cases.

## Supplementary Materials

Supplementary materials can be found at http://www.mdpi.com/1422-0067/19/8/2438/s1.
