# Supplementary material for: Relaxin 2/RXFP1 Signaling Induces Cell Invasion via the β-Catenin Pathway in Endometrial Cancer
Source: Int J Mol Sci. 2018 Aug 18;19(8):2438. doi: 10.3390/ijms19082438 (PMC6121407; doi:10.3390/ijms19082438)
Supplement: Supplementary file 1 [file ijms-19-02438-s001.pdf]

Supplementary Materials: Relaxin 2/RXFP1 Signaling Induces Cell Invasion via the β-Catenin Pathway in Endometrial Cancer

Misaki Fue, Yasuhiro Miki, Kiyoshi Takagi, Chiaki Hashimoto, Nobuo Yaegashi, Takashi Suzuki and Kiyoshi Ito

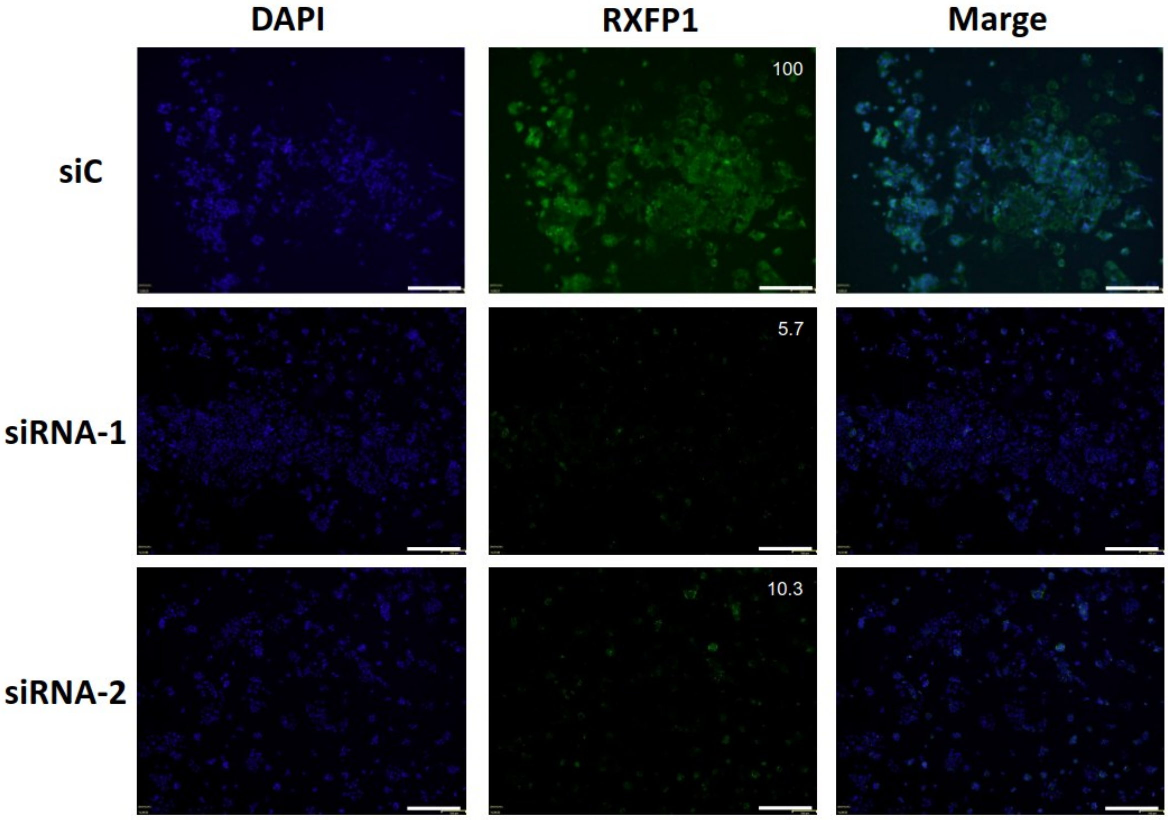

Figure S1. Immunofluorescence staining of RXFP1 after the transfection of HEC-1B with 2 different siRNA molecules. Relative intensity values normalized to the values at the control (siC = 100) are shown. Scale bar, 200 μm.

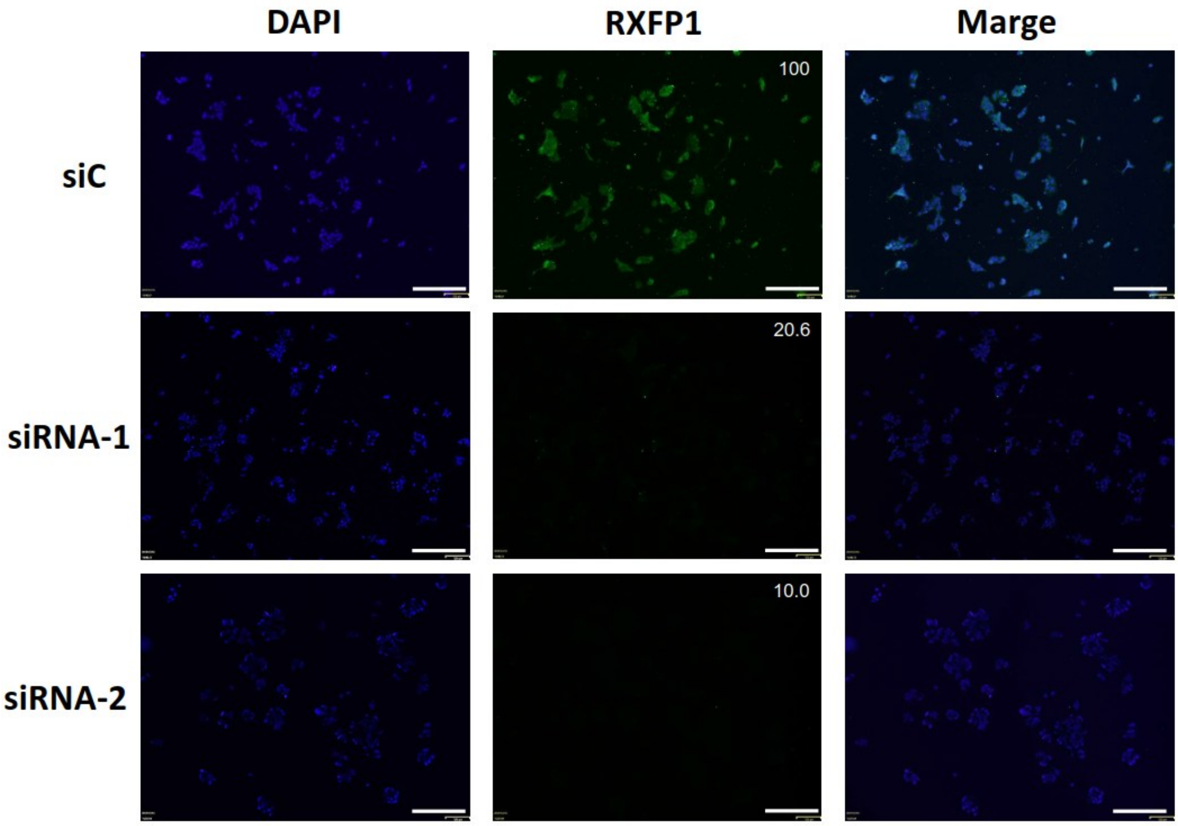

Figure S2. Immunofluorescence staining of RXFP1 after the transfection of Ishikawa with 2 different siRNA molecules. Relative intensity values normalized to the values at the control (siC = 100) are shown. Scale bar, 200 μm.
